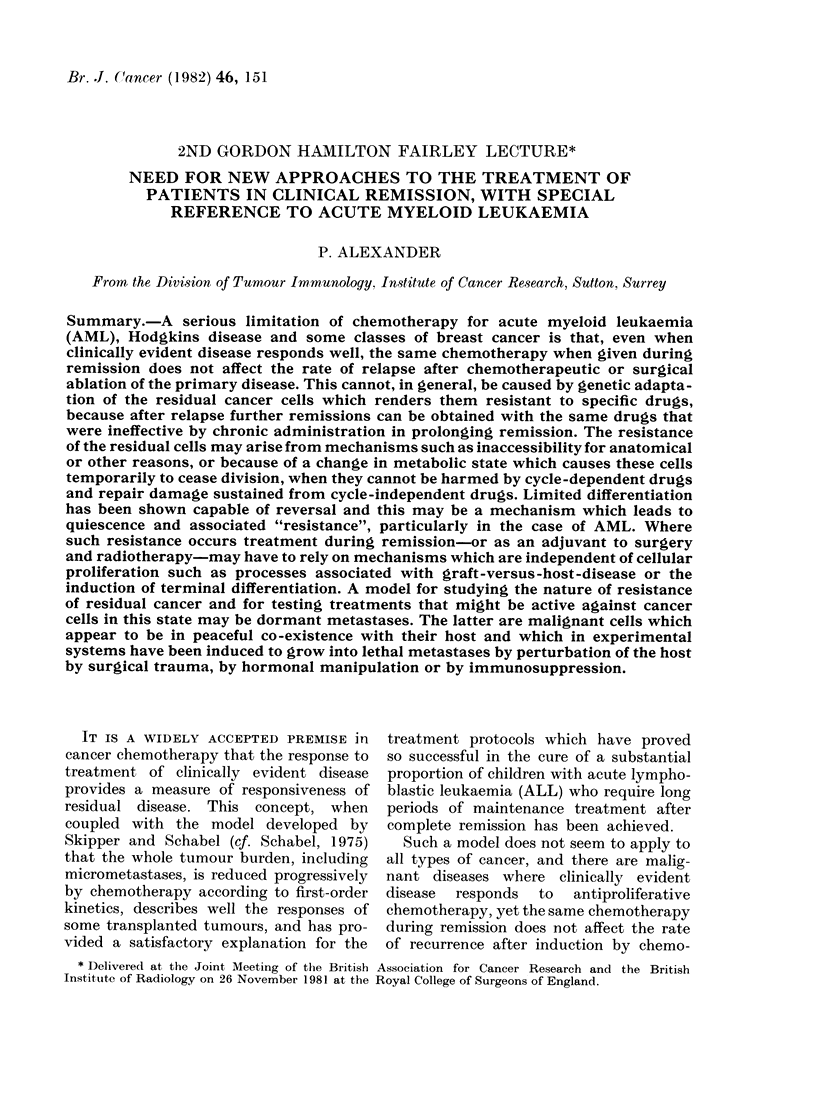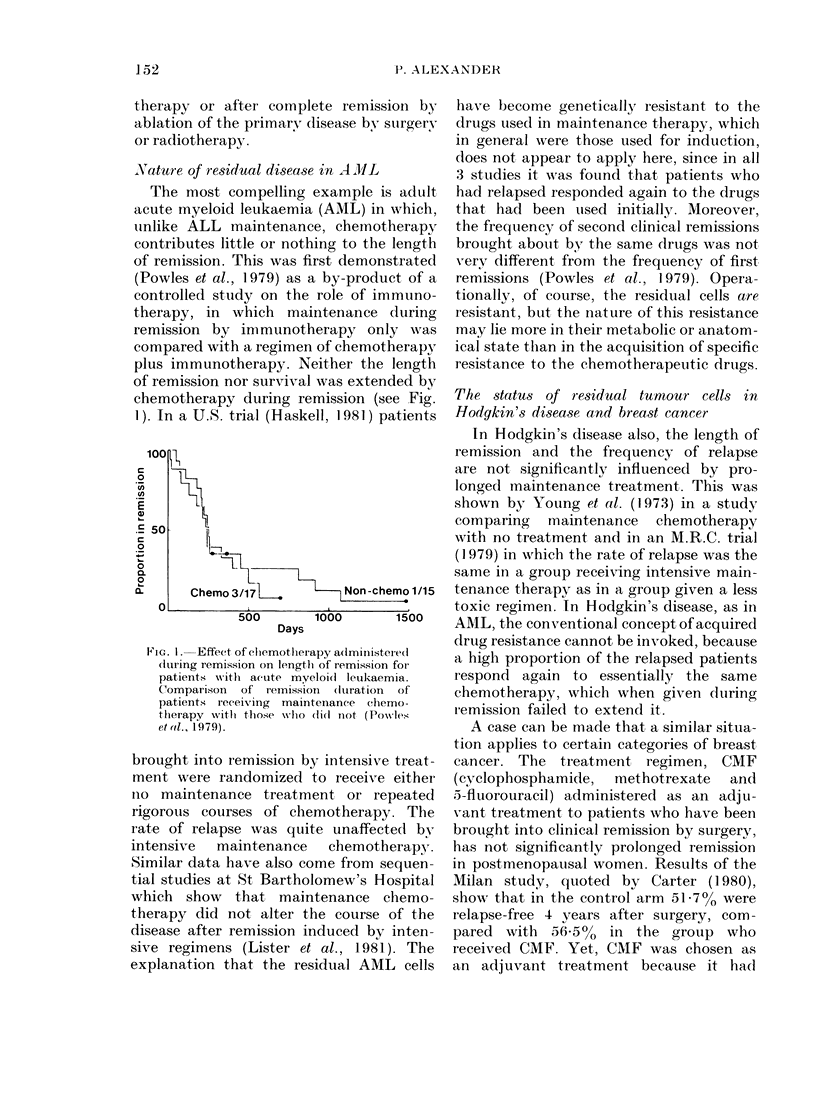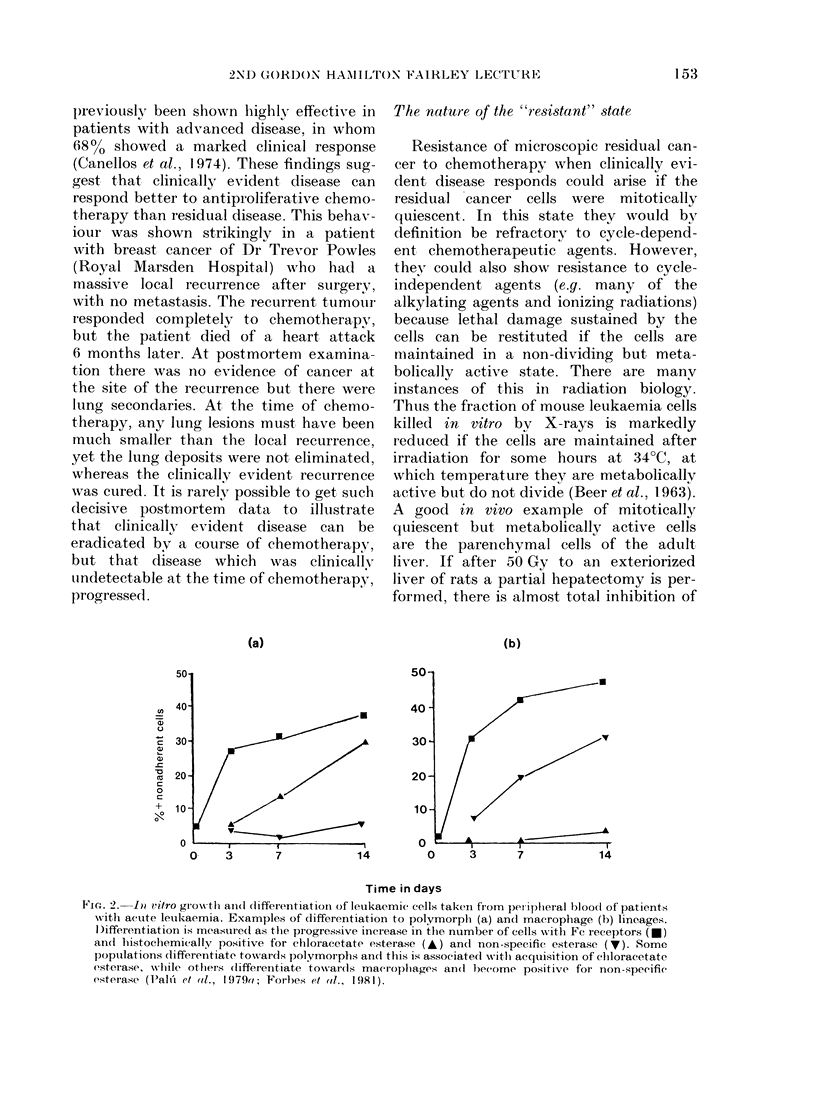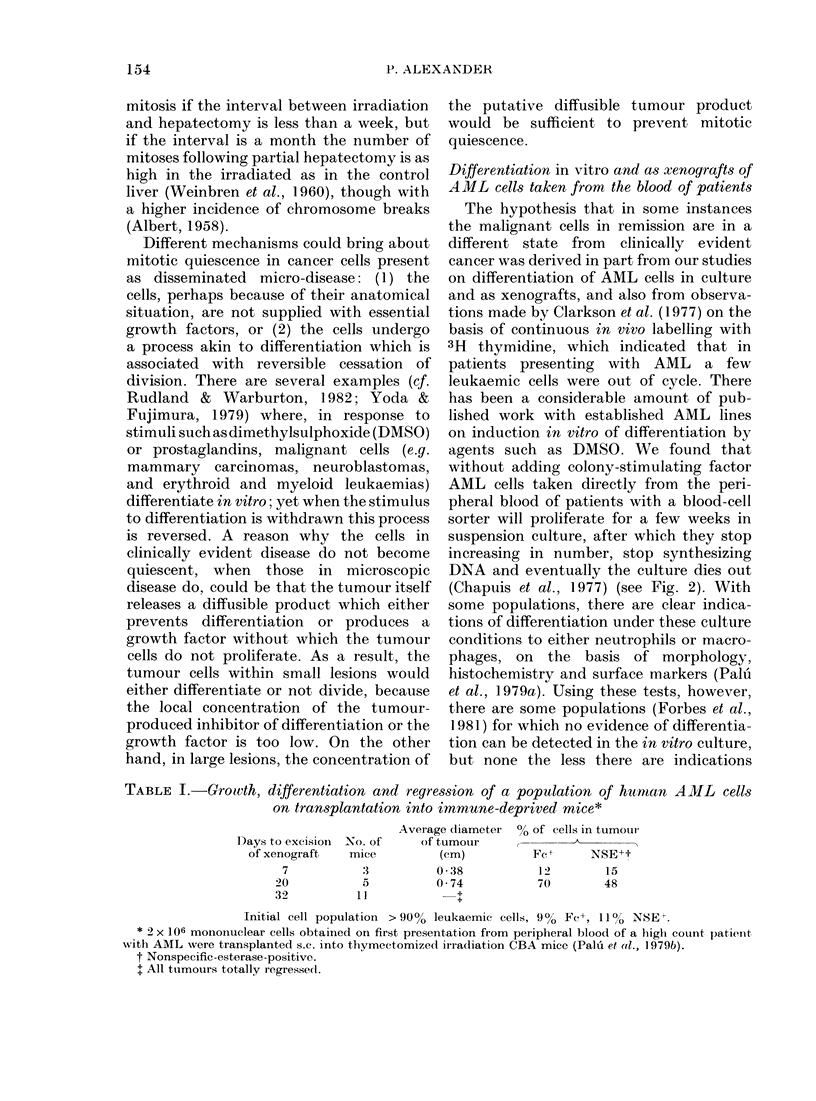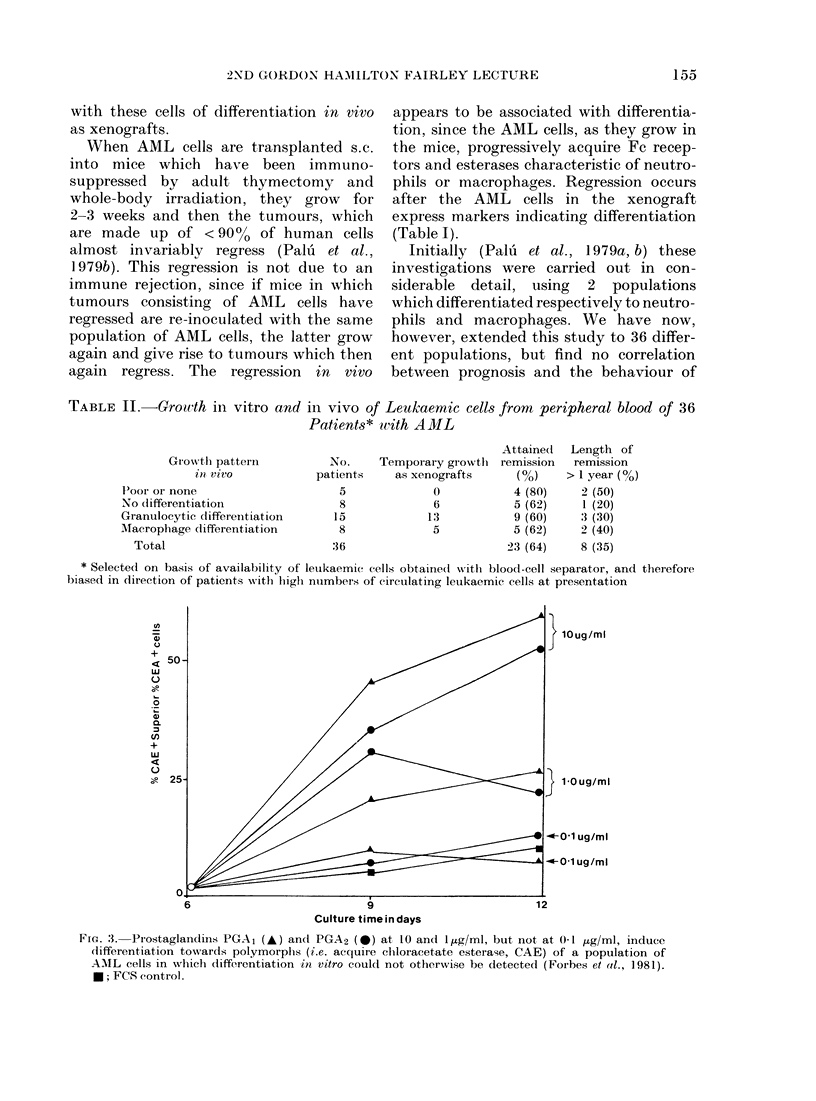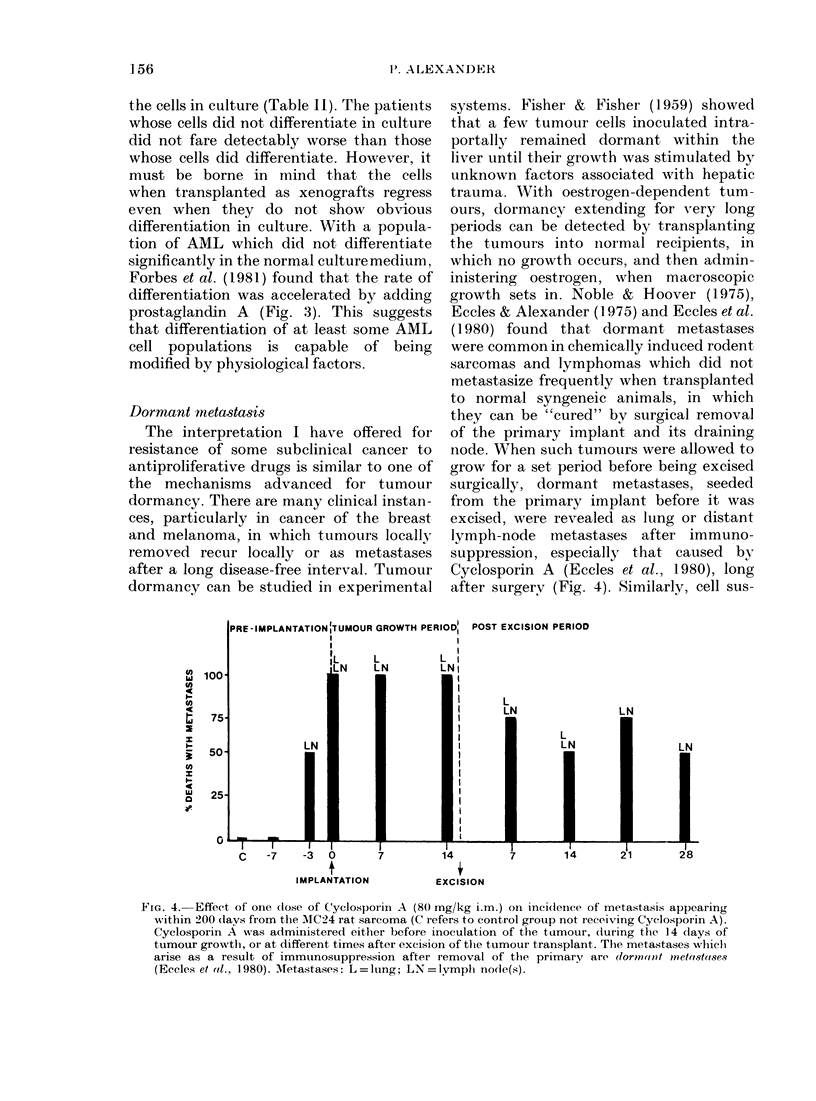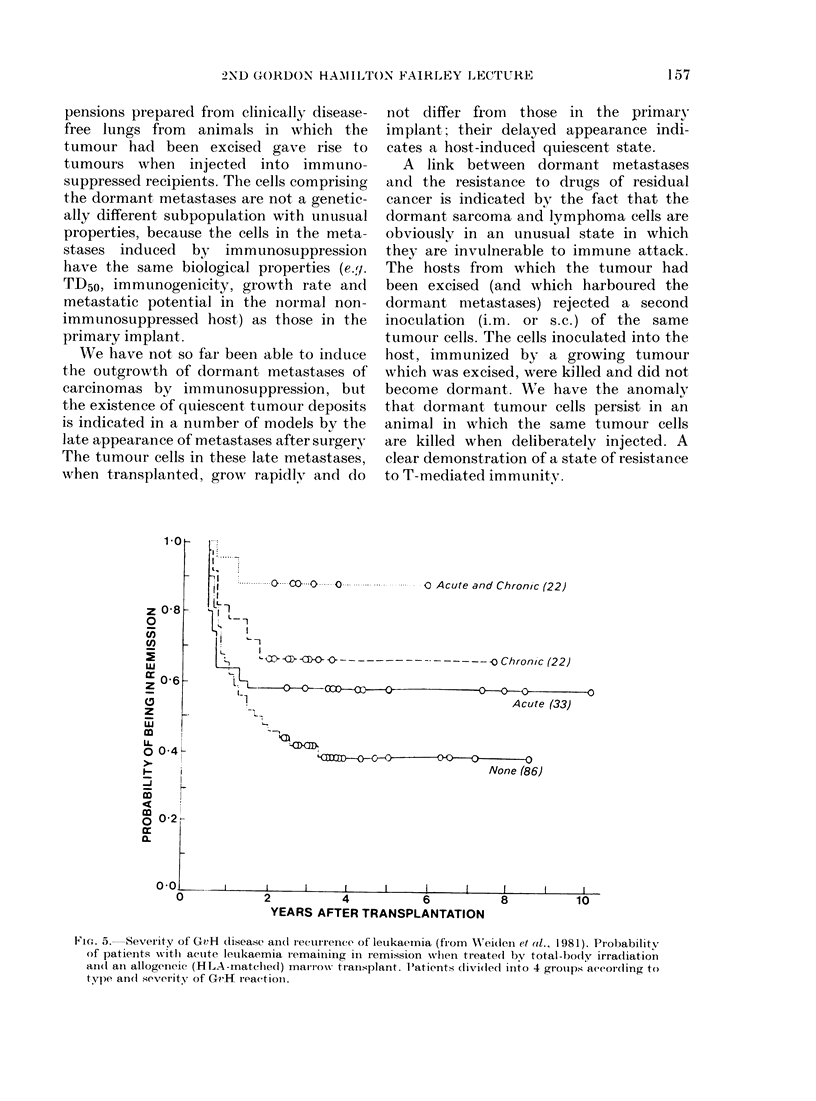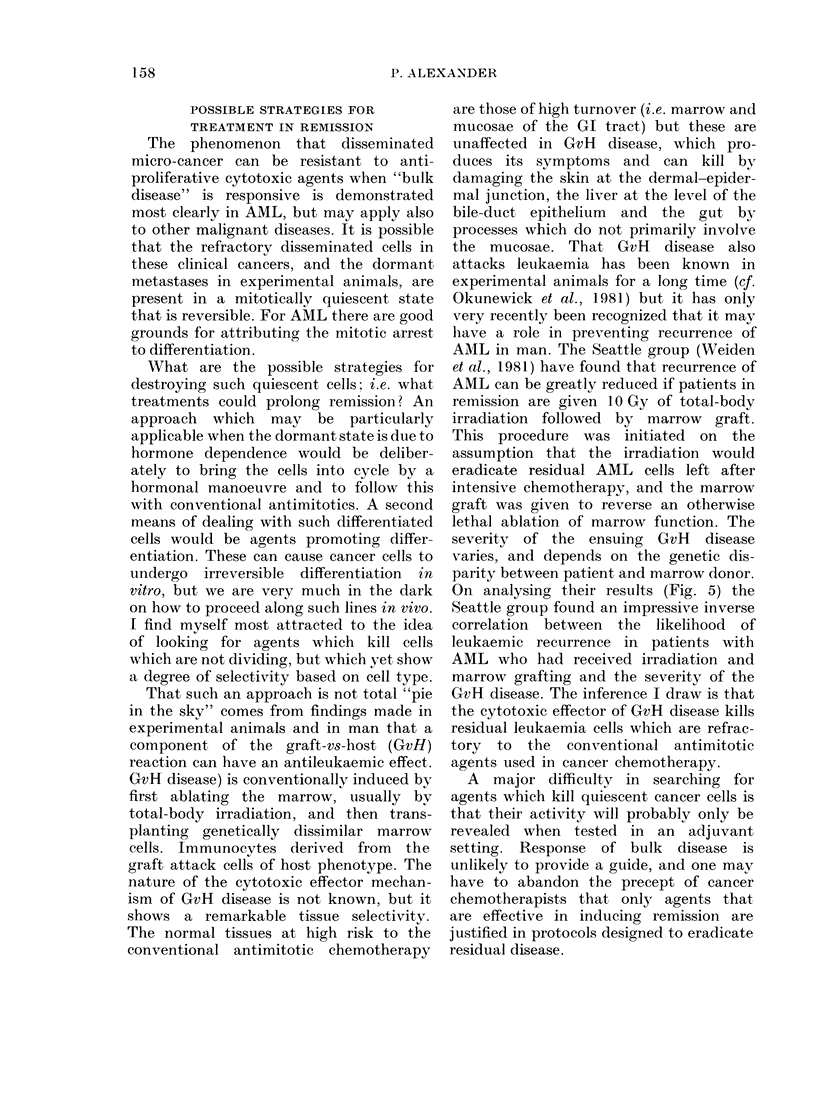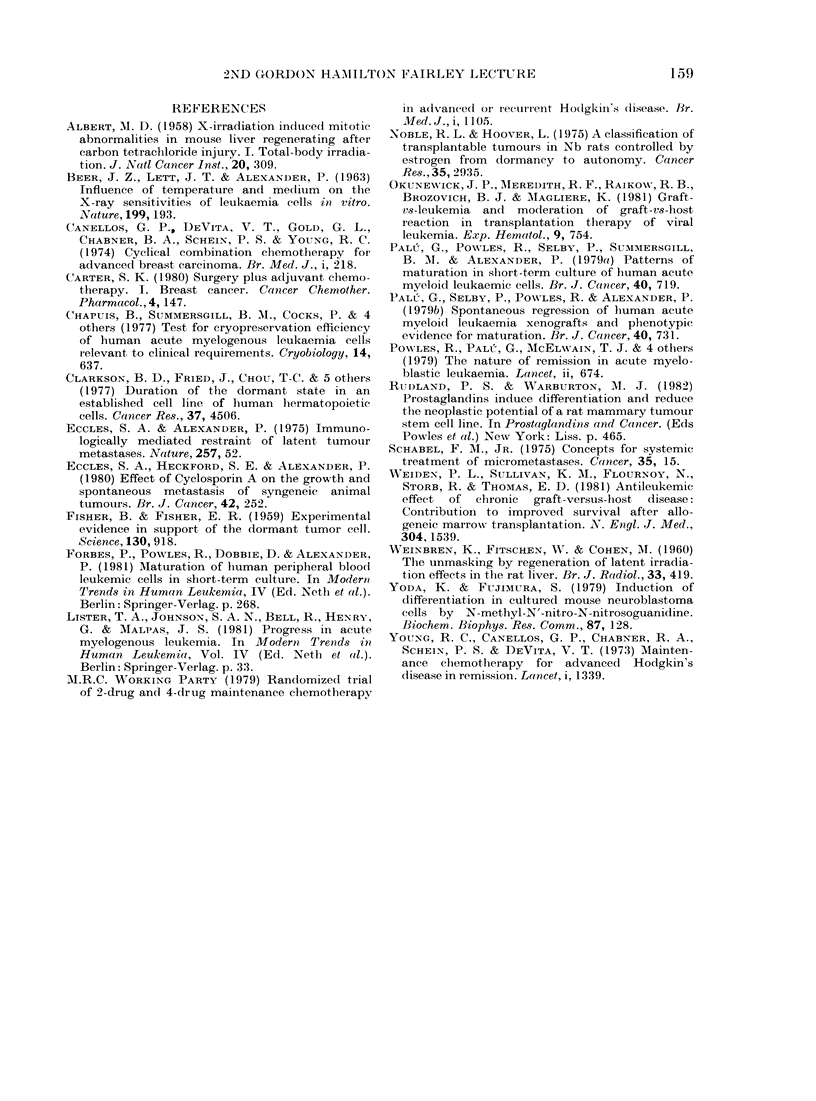# 2nd Gordon Hamilton Fairley Lecture*

**DOI:** 10.1038/bjc.1982.178

**Published:** 1982-08

**Authors:** P. Alexander

## Abstract

A serious limitation of chemotherapy for acute myeloid leukaemia (AML), Hodgkins disease and some classes of breast cancer is that, even when clinically evident disease responds well, the same chemotherapy when given during remission does not affect the rate of relapse after chemotherapeutic or surgical ablation of the primary disease. This cannot, in general, be caused by genetic adaptation of the residual cancer cells which renders them resistant to specific drugs, because after relapse further remissions can be obtained with the same drugs that were ineffective by chronic administration in prolonging remission. The resistance of the residual cells may arise from mechanisms such as inaccessibility for anatomical or other reasons, or because of a change in metabolic state which causes these cells temporarily to cease division, when they cannot be harmed by cycle-dependent drugs and repair damage sustained from cycle-independent drugs. Limited differentiation has been shown capable of reversal and this may be a mechanism which leads to quiescence and associated “resistance”, particularly in the case of AML. Where such resistance occurs treatment during remission—or as an adjuvant to surgery and radiotherapy—may have to rely on mechanisms which are independent of cellular proliferation such as processes associated with graft-versus-host-disease or the induction of terminal differentiation. A model for studying the nature of resistance of residual cancer and for testing treatments that might be active against cancer cells in this state may be dormant metastases. The latter are malignant cells which appear to be in peaceful co-existence with their host and which in experimental systems have been induced to grow into lethal metastases by perturbation of the host by surgical trauma, by hormonal manipulation or by immunosuppression.


Br. J. Cancer (1982) 46, 151

### 2ND GORDON HAMILTON FAIRLEY LECTURE*

### NEED FOR NEW APPROACHES TO THE TREATMENT OF PATIENTS IN CLINICAL REMISSION, WITH SPECIAL REFERENCE TO ACUTE MYELOID LEUKAEMIA

P. ALEXANDER

*From the Division of Tumour Immunology, Institute of Cancer Research, Sutton, Surrey*

**Summary.—A serious limitation of chemotherapy for acute myeloid leukaemia (AML), Hodgkins disease and some classes of breast cancer is that, even when clinically evident disease responds well, the same chemotherapy when given during remission does not affect the rate of relapse after chemotherapeutic or surgical ablation of the primary disease. This cannot, in general, be caused by genetic adaptation of the residual cancer cells which renders them resistant to specific drugs, because after relapse further remissions can be obtained with the same drugs that were ineffective by chronic administration in prolonging remission. The resistance of the residual cells may arise from mechanisms such as inaccessibility for anatomical or other reasons, or because of a change in metabolic state which causes these cells temporarily to cease division, when they cannot be harmed by cycle-dependent drugs and repair damage sustained from cycle-independent drugs. Limited differentiation has been shown capable of reversal and this may be a mechanism which leads to quiescence and associated "resistance", particularly in the case of AML. Where such resistance occurs treatment during remission—or as an adjuvant to surgery and radiotherapy—may have to rely on mechanisms which are independent of cellular proliferation such as processes associated with graft-versus-host-disease or the induction of terminal differentiation. A model for studying the nature of resistance of residual cancer and for testing treatments that might be active against cancer cells in this state may be dormant metastases. The latter are malignant cells which appear to be in peaceful co-existence with their host and which in experimental systems have been induced to grow into lethal metastases by perturbation of the host by surgical trauma, by hormonal manipulation or by immunosuppression.**

IT IS A WIDELY ACCEPTED PREMISE in cancer chemotherapy that the response to treatment of clinically evident disease provides a measure of responsiveness of residual disease. This concept, when coupled with the model developed by Skipper and Schabel (*cf.* Schabel, 1975) that the whole tumour burden, including micrometastases, is reduced progressively by chemotherapy according to first-order kinetics, describes well the responses of some transplanted tumours, and has provided a satisfactory explanation for the treatment protocols which have proved so successful in the cure of a substantial proportion of children with acute lymphoblastic leukaemia (ALL) who require long periods of maintenance treatment after complete remission has been achieved.

Such a model does not seem to apply to all types of cancer, and there are malignant diseases where clinically evident disease responds to antiproliferative chemotherapy, yet the same chemotherapy during remission does not affect the rate of recurrence after induction by chemo-

* Delivered at the Joint Meeting of the British Association for Cancer Research and the British Institute of Radiology on 26 November 1981 at the Royal College of Surgeons of England.

therapy or after complete remission by ablation of the primary disease by surgery or radiotherapy.

*Nature of residual disease in AML*

The most compelling example is adult acute myeloid leukaemia (AML) in which, unlike ALL maintenance, chemotherapy contributes little or nothing to the length of remission. This was first demonstrated (Powles *et al.*, 1979) as a by-product of a controlled study on the role of immunotherapy, in which maintenance during remission by immunotherapy only was compared with a regimen of chemotherapy plus immunotherapy. Neither the length of remission nor survival was extended by chemotherapy during remission (see Fig. 1). In a U.S. trial (Haskell, 1981) patients

FIG. 1.—Effect of chemotherapy administered during remission on length of remission for patients with acute myeloid leukaemia. Comparison of remission duration of patients receiving maintenance chemotherapy with those who did not (Powles *et al.*, 1979).

brought into remission by intensive treatment were randomized to receive either no maintenance treatment or repeated rigorous courses of chemotherapy. The rate of relapse was quite unaffected by intensive maintenance chemotherapy. Similar data have also come from sequential studies at St Bartholomew's Hospital which show that maintenance chemotherapy did not alter the course of the disease after remission induced by intensive regimens (Lister *et al.*, 1981). The explanation that the residual AML cells have become genetically resistant to the drugs used in maintenance therapy, which in general were those used for induction, does not appear to apply here, since in all 3 studies it was found that patients who had relapsed responded again to the drugs that had been used initially. Moreover, the frequency of second clinical remissions brought about by the same drugs was not very different from the frequency of first remissions (Powles *et al.*, 1979). Operationally, of course, the residual cells *are* resistant, but the nature of this resistance may lie more in their metabolic or anatomical state than in the acquisition of specific resistance to the chemotherapeutic drugs.

*The status of residual tumour cells in Hodgkin's disease and breast cancer*

In Hodgkin's disease also, the length of remission and the frequency of relapse are not significantly influenced by prolonged maintenance treatment. This was shown by Young *et al.* (1973) in a study comparing maintenance chemotherapy with no treatment and in an M.R.C. trial (1979) in which the rate of relapse was the same in a group receiving intensive maintenance therapy as in a group given a less toxic regimen. In Hodgkin's disease, as in AML, the conventional concept of acquired drug resistance cannot be invoked, because a high proportion of the relapsed patients respond again to essentially the same chemotherapy, which when given during remission failed to extend it.

A case can be made that a similar situation applies to certain categories of breast cancer. The treatment regimen, CMF (cyclophosphamide, methotrexate and 5-fluorouracil) administered as an adjuvant treatment to patients who have been brought into clinical remission by surgery, has not significantly prolonged remission in postmenopausal women. Results of the Milan study, quoted by Carter (1980), show that in the control arm 51·7% were relapse-free 4 years after surgery, compared with 56·5% in the group who received CMF. Yet, CMF was chosen as an adjuvant treatment because it had previously been shown highly effective in patients with advanced disease, in whom 68% showed a marked clinical response (Canellos *et al.*, 1974). These findings suggest that clinically evident disease can respond better to antiproliferative chemotherapy than residual disease. This behaviour was shown strikingly in a patient with breast cancer of Dr Trevor Powles (Royal Marsden Hospital) who had a massive local recurrence after surgery, with no metastasis. The recurrent tumour responded completely to chemotherapy, but the patient died of a heart attack 6 months later. At postmortem examination there was no evidence of cancer at the site of the recurrence but there were lung secondaries. At the time of chemotherapy, any lung lesions must have been much smaller than the local recurrence, yet the lung deposits were not eliminated, whereas the clinically evident recurrence was cured. It is rarely possible to get such decisive postmortem data to illustrate that clinically evident disease can be eradicated by a course of chemotherapy, but that disease which was clinically undetectable at the time of chemotherapy, progressed.

### *The nature of the "resistant" state*

Resistance of microscopic residual cancer to chemotherapy when clinically evident disease responds could arise if the residual cancer cells were mitotically quiescent. In this state they would by definition be refractory to cycle-dependent chemotherapeutic agents. However, they could also show resistance to cycle-independent agents (*e.g.* many of the alkylating agents and ionizing radiations) because lethal damage sustained by the cells can be restituted if the cells are maintained in a non-dividing but metabolically active state. There are many instances of this in radiation biology. Thus the fraction of mouse leukaemia cells killed *in vitro* by X-rays is markedly reduced if the cells are maintained after irradiation for some hours at 34°C, at which temperature they are metabolically active but do not divide (Beer *et al.*, 1963). A good *in vivo* example of mitotically quiescent but metabolically active cells are the parenchymal cells of the adult liver. If after 50 Gy to an exteriorized liver of rats a partial hepatectomy is performed, there is almost total inhibition of

FIG. 2.—*In vitro* growth and differentiation of leukaemic cells taken from peripheral blood of patients with acute leukaemia. Examples of differentiation to polymorph (a) and macrophage (b) lineages. Differentiation is measured as the progressive increase in the number of cells with Fc receptors (■) and histochemically positive for chloracetate esterase (▲) and non-specific esterase (▼). Some populations differentiate towards polymorphs and this is associated with acquisition of chloracetate esterase, while others differentiate towards macrophages and become positive for non-specific esterase (Palú *et al.*, 1979*a*; Forbes *et al.*, 1981).

mitosis if the interval between irradiation and hepatectomy is less than a week, but if the interval is a month the number of mitoses following partial hepatectomy is as high in the irradiated as in the control liver (Weinbren *et al.*, 1960), though with a higher incidence of chromosome breaks (Albert, 1958).

Different mechanisms could bring about mitotic quiescence in cancer cells present as disseminated micro-disease: (1) the cells, perhaps because of their anatomical situation, are not supplied with essential growth factors, or (2) the cells undergo a process akin to differentiation which is associated with reversible cessation of division. There are several examples (*cf.* Rudland & Warburton, 1982; Yoda & Fujimura, 1979) where, in response to stimuli such as dimethylsulphoxide (DMSO) or prostaglandins, malignant cells (*e.g.* mammary carcinomas, neuroblastomas, and erythroid and myeloid leukaemias) differentiate *in vitro*; yet when the stimulus to differentiation is withdrawn this process is reversed. A reason why the cells in clinically evident disease do not become quiescent, when those in microscopic disease do, could be that the tumour itself releases a diffusible product which either prevents differentiation or produces a growth factor without which the tumour cells do not proliferate. As a result, the tumour cells within small lesions would either differentiate or not divide, because the local concentration of the tumour-produced inhibitor of differentiation or the growth factor is too low. On the other hand, in large lesions, the concentration of the putative diffusible tumour product would be sufficient to prevent mitotic quiescence.

*Differentiation* in vitro *and as xenografts of AML cells taken from the blood of patients*

The hypothesis that in some instances the malignant cells in remission are in a different state from clinically evident cancer was derived in part from our studies on differentiation of AML cells in culture and as xenografts, and also from observations made by Clarkson *et al.* (1977) on the basis of continuous *in vivo* labelling with $^{3}$H thymidine, which indicated that in patients presenting with AML a few leukaemic cells were out of cycle. There has been a considerable amount of published work with established AML lines on induction *in vitro* of differentiation by agents such as DMSO. We found that without adding colony-stimulating factor AML cells taken directly from the peripheral blood of patients with a blood-cell sorter will proliferate for a few weeks in suspension culture, after which they stop increasing in number, stop synthesizing DNA and eventually the culture dies out (Chapuis *et al.*, 1977) (see Fig. 2). With some populations, there are clear indications of differentiation under these culture conditions to either neutrophils or macrophages, on the basis of morphology, histochemistry and surface markers (Palú *et al.*, 1979*a*). Using these tests, however, there are some populations (Forbes *et al.*, 1981) for which no evidence of differentiation can be detected in the *in vitro* culture, but none the less there are indications

TABLE I.—*Growth, differentiation and regression of a population of human AML cells on transplantation into immune-deprived mice**

| Days to excision of xenograft | No. of mice | Average diameter of tumour (cm) | % of cells in tumour: Fc$^{+}$ | % of cells in tumour: NSE$^{+}$† |
|---|---|---|---|---|
| 7 | 3 | 0·38 | 12 | 15 |
| 20 | 5 | 0·74 | 70 | 48 |
| 32 | 11 | —‡ | | |

Initial cell population >90% leukaemic cells, 9% Fc$^{+}$, 11% NSE$^{+}$.

* $2\times10^{6}$ mononuclear cells obtained on first presentation from peripheral blood of a high count patient with AML were transplanted s.c. into thymectomized irradiation CBA mice (Palú *et al.*, 1979*b*).

† Nonspecific-esterase-positive.

‡ All tumours totally regressed.

with these cells of differentiation *in vivo* as xenografts.

When AML cells are transplanted s.c. into mice which have been immunosuppressed by adult thymectomy and whole-body irradiation, they grow for 2–3 weeks and then the tumours, which are made up of < 90% of human cells almost invariably regress (Palú *et al.*, 1979*b*). This regression is not due to an immune rejection, since if mice in which tumours consisting of AML cells have regressed are re-inoculated with the same population of AML cells, the latter grow again and give rise to tumours which then again regress. The regression *in vivo* appears to be associated with differentiation, since the AML cells, as they grow in the mice, progressively acquire Fc receptors and esterases characteristic of neutrophils or macrophages. Regression occurs after the AML cells in the xenograft express markers indicating differentiation (Table I).

Initially (Palú *et al.*, 1979*a, b*) these investigations were carried out in considerable detail, using 2 populations which differentiated respectively to neutrophils and macrophages. We have now, however, extended this study to 36 different populations, but find no correlation between prognosis and the behaviour of

TABLE II.—*Growth* in vitro *and* in vivo *of Leukaemic cells from peripheral blood of* 36 *Patients* with AML*

| Growth pattern *in vivo* | No. patients | Temporary growth as xenografts | Attained remission (%) | Length of remission > 1 year (%) |
|---|---|---|---|---|
| Poor or none | 5 | 0 | 4 (80) | 2 (50) |
| No differentiation | 8 | 6 | 5 (62) | 1 (20) |
| Granulocytic differentiation | 15 | 13 | 9 (60) | 3 (30) |
| Macrophage differentiation | 8 | 5 | 5 (62) | 2 (40) |
| Total | 36 | | 23 (64) | 8 (35) |

* Selected on basis of availability of leukaemic cells obtained with blood-cell separator, and therefore biased in direction of patients with high numbers of circulating leukaemic cells at presentation

FIG. 3.—Prostaglandins $PGA_1$ (▲) and $PGA_2$ (●) at 10 and 1μg/ml, but not at 0·1 μg/ml, induce differentiation towards polymorphs (*i.e.* acquire chloracetate esterase, CAE) of a population of AML cells in which differentiation *in vitro* could not otherwise be detected (Forbes *et al.*, 1981). ■; FCS control.

the cells in culture (Table II). The patients whose cells did not differentiate in culture did not fare detectably worse than those whose cells did differentiate. However, it must be borne in mind that the cells when transplanted as xenografts regress even when they do not show obvious differentiation in culture. With a population of AML which did not differentiate significantly in the normal culture medium, Forbes *et al.* (1981) found that the rate of differentiation was accelerated by adding prostaglandin A (Fig. 3). This suggests that differentiation of at least some AML cell populations is capable of being modified by physiological factors.

*Dormant metastasis*

The interpretation I have offered for resistance of some subclinical cancer to antiproliferative drugs is similar to one of the mechanisms advanced for tumour dormancy. There are many clinical instances, particularly in cancer of the breast and melanoma, in which tumours locally removed recur locally or as metastases after a long disease-free interval. Tumour dormancy can be studied in experimental systems. Fisher & Fisher (1959) showed that a few tumour cells inoculated intraportally remained dormant within the liver until their growth was stimulated by unknown factors associated with hepatic trauma. With oestrogen-dependent tumours, dormancy extending for very long periods can be detected by transplanting the tumours into normal recipients, in which no growth occurs, and then administering oestrogen, when macroscopic growth sets in. Noble & Hoover (1975), Eccles & Alexander (1975) and Eccles *et al.* (1980) found that dormant metastases were common in chemically induced rodent sarcomas and lymphomas which did not metastasize frequently when transplanted to normal syngeneic animals, in which they can be "cured" by surgical removal of the primary implant and its draining node. When such tumours were allowed to grow for a set period before being excised surgically, dormant metastases, seeded from the primary implant before it was excised, were revealed as lung or distant lymph-node metastases after immunosuppression, especially that caused by Cyclosporin A (Eccles *et al.*, 1980), long after surgery (Fig. 4). Similarly, cell sus-

FIG. 4.—Effect of one dose of Cyclosporin A (80 mg/kg i.m.) on incidence of metastasis appearing within 200 days from the MC24 rat sarcoma (C refers to control group not receiving Cyclosporin A). Cyclosporin A was administered either before inoculation of the tumour, during the 14 days of tumour growth, or at different times after excision of the tumour transplant. The metastases which arise as a result of immunosuppression after removal of the primary are *dormant metastases* (Eccles *et al.*, 1980). Metastases: L = lung; LN = lymph node(s).

pensions prepared from clinically disease-free lungs from animals in which the tumour had been excised gave rise to tumours when injected into immunosuppressed recipients. The cells comprising the dormant metastases are not a genetically different subpopulation with unusual properties, because the cells in the metastases induced by immunosuppression have the same biological properties (*e.g.* $TD_{50}$, immunogenicity, growth rate and metastatic potential in the normal non-immunosuppressed host) as those in the primary implant.

We have not so far been able to induce the outgrowth of dormant metastases of carcinomas by immunosuppression, but the existence of quiescent tumour deposits is indicated in a number of models by the late appearance of metastases after surgery The tumour cells in these late metastases, when transplanted, grow rapidly and do not differ from those in the primary implant; their delayed appearance indicates a host-induced quiescent state.

A link between dormant metastases and the resistance to drugs of residual cancer is indicated by the fact that the dormant sarcoma and lymphoma cells are obviously in an unusual state in which they are invulnerable to immune attack. The hosts from which the tumour had been excised (and which harboured the dormant metastases) rejected a second inoculation (i.m. or s.c.) of the same tumour cells. The cells inoculated into the host, immunized by a growing tumour which was excised, were killed and did not become dormant. We have the anomaly that dormant tumour cells persist in an animal in which the same tumour cells are killed when deliberately injected. A clear demonstration of a state of resistance to T-mediated immunity.

FIG. 5.—Severity of GvH disease and recurrence of leukaemia (from Weiden *et al.*, 1981). Probability of patients with acute leukaemia remaining in remission when treated by total-body irradiation and an allogeneic (HLA-matched) marrow transplant. Patients divided into 4 groups according to type and severity of GvH reaction.

### POSSIBLE STRATEGIES FOR TREATMENT IN REMISSION

The phenomenon that disseminated micro-cancer can be resistant to anti-proliferative cytotoxic agents when "bulk disease" is responsive is demonstrated most clearly in AML, but may apply also to other malignant diseases. It is possible that the refractory disseminated cells in these clinical cancers, and the dormant metastases in experimental animals, are present in a mitotically quiescent state that is reversible. For AML there are good grounds for attributing the mitotic arrest to differentiation.

What are the possible strategies for destroying such quiescent cells; *i.e.* what treatments could prolong remission? An approach which may be particularly applicable when the dormant state is due to hormone dependence would be deliberately to bring the cells into cycle by a hormonal manoeuvre and to follow this with conventional antimitotics. A second means of dealing with such differentiated cells would be agents promoting differentiation. These can cause cancer cells to undergo irreversible differentiation *in vitro*, but we are very much in the dark on how to proceed along such lines *in vivo*. I find myself most attracted to the idea of looking for agents which kill cells which are not dividing, but which yet show a degree of selectivity based on cell type.

That such an approach is not total "pie in the sky" comes from findings made in experimental animals and in man that a component of the graft-*vs*-host (G*vH*) reaction can have an antileukaemic effect. G*v*H disease) is conventionally induced by first ablating the marrow, usually by total-body irradiation, and then transplanting genetically dissimilar marrow cells. Immunocytes derived from the graft attack cells of host phenotype. The nature of the cytotoxic effector mechanism of G*v*H disease is not known, but it shows a remarkable tissue selectivity. The normal tissues at high risk to the conventional antimitotic chemotherapy are those of high turnover (*i.e.* marrow and mucosae of the GI tract) but these are unaffected in G*v*H disease, which produces its symptoms and can kill by damaging the skin at the dermal–epidermal junction, the liver at the level of the bile-duct epithelium and the gut by processes which do not primarily involve the mucosae. That G*v*H disease also attacks leukaemia has been known in experimental animals for a long time (*cf.* Okunewick *et al.*, 1981) but it has only very recently been recognized that it may have a role in preventing recurrence of AML in man. The Seattle group (Weiden *et al.*, 1981) have found that recurrence of AML can be greatly reduced if patients in remission are given 10 Gy of total-body irradiation followed by marrow graft. This procedure was initiated on the assumption that the irradiation would eradicate residual AML cells left after intensive chemotherapy, and the marrow graft was given to reverse an otherwise lethal ablation of marrow function. The severity of the ensuing G*v*H disease varies, and depends on the genetic disparity between patient and marrow donor. On analysing their results (Fig. 5) the Seattle group found an impressive inverse correlation between the likelihood of leukaemic recurrence in patients with AML who had received irradiation and marrow grafting and the severity of the G*v*H disease. The inference I draw is that the cytotoxic effector of G*v*H disease kills residual leukaemia cells which are refractory to the conventional antimitotic agents used in cancer chemotherapy.

A major difficulty in searching for agents which kill quiescent cancer cells is that their activity will probably only be revealed when tested in an adjuvant setting. Response of bulk disease is unlikely to provide a guide, and one may have to abandon the precept of cancer chemotherapists that only agents that are effective in inducing remission are justified in protocols designed to eradicate residual disease.

REFERENCES

ALBERT, M. D. (1958) X-irradiation induced mitotic abnormalities in mouse liver regenerating after carbon tetrachloride injury. I. Total-body irradiation. *J. Natl Cancer Inst.*, **20**, 309.

BEER, J. Z., LETT, J. T. & ALEXANDER, P. (1963) Influence of temperature and medium on the X-ray sensitivities of leukaemia cells *in vitro*. *Nature*, **199**, 193.

CANELLOS, G. P., DEVITA, V. T., GOLD, G. L., CHABNER, B. A., SCHEIN, P. S. & YOUNG, R. C. (1974) Cyclical combination chemotherapy for advanced breast carcinoma. *Br. Med. J.*, i, 218.

CARTER, S. K. (1980) Surgery plus adjuvant chemotherapy. I. Breast cancer. *Cancer Chemother. Pharmacol.*, **4**, 147.

CHAPUIS, B., SUMMERSGILL, B. M., COCKS, P. & 4 others (1977) Test for cryopreservation efficiency of human acute myelogenous leukaemia cells relevant to clinical requirements. *Cryobiology*, **14**, 637.

CLARKSON, B. D., FRIED, J., CHOU, T-C. & 5 others (1977) Duration of the dormant state in an established cell line of human hermatopoietic cells. *Cancer Res.*, **37**, 4506.

ECCLES, S. A. & ALEXANDER, P. (1975) Immunologically mediated restraint of latent tumour metastases. *Nature*, **257**, 52.

ECCLES, S. A., HECKFORD, S. E. & ALEXANDER, P. (1980) Effect of Cyclosporin A on the growth and spontaneous metastasis of syngeneic animal tumours. *Br. J. Cancer*, **42**, 252.

FISHER, B. & FISHER, E. R. (1959) Experimental evidence in support of the dormant tumor cell. *Science*, **130**, 918.

FORBES, P., POWLES, R., DOBBIE, D. & ALEXANDER, P. (1981) Maturation of human peripheral blood leukemic cells in short-term culture. In *Modern Trends in Human Leukemia*, IV (Ed. Neth *et al.*). Berlin: Springer-Verlag. p. 268.

LISTER, T. A., JOHNSON, S. A. N., BELL, R., HENRY, G. & MALPAS, J. S. (1981) Progress in acute myelogenous leukemia. In *Modern Trends in Human Leukemia*, Vol. IV (Ed. Neth *et al.*). Berlin: Springer-Verlag. p. 33.

M.R.C. WORKING PARTY (1979) Randomized trial of 2-drug and 4-drug maintenance chemotherapy in advanced or recurrent Hodgkin's disease. *Br. Med. J.*, i, 1105.

NOBLE, R. L. & HOOVER, L. (1975) A classification of transplantable tumours in Nb rats controlled by estrogen from dormancy to autonomy. *Cancer Res.*, **35**, 2935.

OKUNEWICK, J. P., MEREDITH, R. F., RAIKOW, R. B., BROZOVICH, B. J. & MAGLIERE, K. (1981) Graft-*vs*-leukemia and moderation of graft-*vs*-host reaction in transplantation therapy of viral leukemia. *Exp. Hematol.*, **9**, 754.

PALÚ, G., POWLES, R., SELBY, P., SUMMERSGILL, B. M. & ALEXANDER, P. (1979*a*) Patterns of maturation in short-term culture of human acute myeloid leukaemic cells. *Br. J. Cancer*, **40**, 719.

PALÚ, G., SELBY, P., POWLES, R. & ALEXANDER, P. (1979*b*) Spontaneous regression of human acute myeloid leukaemia xenografts and phenotypic evidence for maturation. *Br. J. Cancer*, **40**, 731.

POWLES, R., PALÚ, G., MCELWAIN, T. J. & 4 others (1979) The nature of remission in acute myeloblastic leukaemia. *Lancet*, ii, 674.

RUDLAND, P. S. & WARBURTON, M. J. (1982) Prostaglandins induce differentiation and reduce the neoplastic potential of a rat mammary tumour stem cell line. In *Prostaglandins and Cancer*. (Eds Powles *et al.*) New York: Liss. p. 465.

SCHABEL, F. M., JR. (1975) Concepts for systemic treatment of micrometastases. *Cancer*, **35**, 15.

WEIDEN, P. L., SULLIVAN, K. M., FLOURNOY, N., STORB, R. & THOMAS, E. D. (1981) Antileukemic effect of chronic graft-versus-host disease: Contribution to improved survival after allogeneic marrow transplantation. *N. Engl. J. Med.*, **304**, 1539.

WEINBREN, K., FITSCHEN, W. & COHEN, M. (1960) The unmasking by regeneration of latent irradiation effects in the rat liver. *Br. J. Radiol.*, **33**, 419.

YODA, K. & FUJIMURA, S. (1979) Induction of differentiation in cultured mouse neuroblastoma cells by N-methyl-N′-nitro-N-nitrosoguanidine. *Biochem. Biophys. Res. Comm.*, **87**, 128.

YOUNG, R. C., CANELLOS, G. P., CHABNER, R. A., SCHEIN, P. S. & DEVITA, V. T. (1973) Maintenance chemotherapy for advanced Hodgkin's disease in remission. *Lancet*, i, 1339.